# Connectivity as a Predictor of Responsiveness to Transcranial Direct Current Stimulation in People with Stroke: Protocol for a Double-Blind Randomized Controlled Trial

**DOI:** 10.2196/10848

**Published:** 2018-10-18

**Authors:** Ellana Welsby, Michael Ridding, Susan Hillier, Brenton Hordacre

**Affiliations:** 1 The Sansom Institute for Health Research School of Health Sciences University of South Australia Adelaide Australia; 2 Neuromotor Plasticity and Development Group Adelaide Medical School University of Adelaide Adelaide Australia

**Keywords:** electroencephalography, magnetic resonance imaging, rehabilitation, stroke, transcranial direct current stimulation, upper limb

## Abstract

**Background:**

Stroke can have devastating consequences for an individual’s quality of life. Interventions capable of enhancing response to therapy would be highly valuable to the field of neurological rehabilitation. One approach is to use noninvasive brain stimulation techniques, such as transcranial direct current stimulation, to induce a neuroplastic response. When delivered in combination with rehabilitation exercises, there is some evidence that transcranial direct current stimulation is beneficial. However, responses to stimulation are highly variable. Therefore biomarkers predictive of response to stimulation would be valuable to help select appropriate people for this potentially beneficial treatment.

**Objective:**

The objective of this study is to investigate connectivity of the stimulation target, the ipsilesional motor cortex, as a biomarker predictive of response to anodal transcranial direct current stimulation in people with stroke.

**Methods:**

This study is a double blind, randomized controlled trial (RCT), with two parallel groups. A total of 68 participants with first ever ischemic stroke with motor impairment will undertake a two week (14 session) treatment for upper limb function (Graded Repetitive Arm Supplementary Program; GRASP). Participants will be randomized 2:1 to active:sham treatment groups. Those in the active treatment group will receive anodal transcranial direct current stimulation to the ipsilesional motor cortex at the start of each GRASP session. Those allocated to the sham treatment group will receive sham transcranial direct current stimulation. Behavioural assessments of upper limb function will be performed at baseline, post treatment, 1 month follow-up and 3 months follow-up. Neurophysiological assessments will include magnetic resonance imaging (MRI), electroencephalography (EEG) and transcranial magnetic stimulation (TMS) and will be performed at baseline, post treatment, 1 month follow-up (EEG and TMS only) and 3 months follow-up (EEG and TMS only).

**Results:**

Participants will be recruited between March 2018 and December 2018, with experimental testing concluding in March 2019.

**Conclusions:**

Identifying a biomarker predictive of response to transcranial direct current stimulation would greatly assist clinical utility of this novel treatment approach.

**Trial Registration:**

Australia New Zealand Clinical Trials Registry ACTRN12618000443291; https://www.anzctr.org.au/Trial/Registration/TrialReview.aspx?ACTRN=12618000443291 (Archived by WebCite at http://www.webcitation.org/737QOXXxt)

**Registered Report Identifier:**

RR1-10.2196/10848

## Introduction

Globally, stroke is a leading cause of death and disability. According to the World Health Organization, there were 6.7 million stroke-related deaths in 2012, with 33 million stroke survivors living with persistent disability, requiring long-term care and secondary prevention measures [1]. A stroke affecting the sensorimotor network can lead to behavioral impairments, restricting the capacity to perform various activities of daily living. As a result, many stroke survivors require multidisciplinary rehabilitation to help restore function [2]. Despite lengthy periods of rehabilitation, significant impairments often remain, suggesting there is a need to do more to assist survivors of stroke.

Restitution of upper limb function following stroke is important to improve capacity to undertake activities of daily living and enhance quality of life. Underpinning functional restitution is a process known as neuroplasticity where both structure and function of the surviving brain tissue can change to optimize behavior. Research indicates there may be a time-limited window of enhanced neuroplasticity following stroke [3,4]. This period of enhanced neuroplasticity following stroke has many similarities to those that occur during development where the brain is highly plastic and rapid learning occurs [3]. Delivering rehabilitative therapies during this time may provide an opportunity for a more complete recovery. It is generally thought that this period of enhanced neuroplasticity occurs early after stroke [3,4]. In support of this, behavioral evidence indicates that therapy delivered early after stroke may be more effective than that delivered later [3,5,6]. Furthermore, consensus statements suggest that delayed initiation of rehabilitation is associated with poor outcomes and longer hospital stay [7,8]. However, it should be noted that while early therapeutic intervention may be more effective, recovery long after stroke remains possible. In support of this, there is good evidence indicating that constraint induced movement therapy is capable of improving function months or years after the initial stroke [9-11], suggesting the window for recovery may never really close [3].

One interesting approach to stroke rehabilitation is to attempt to reestablish a period of enhanced neuroplasticity to boost the effects of therapy in people with stroke. The potential for a more complete functional recovery by re-establishing a period of enhanced neuroplasticity was recently shown in an animal model of stroke [12]. In this study, Zeiler et al [12] demonstrated that a second ischemic event reopened a period of heightened response to training, facilitating recovery from the initial stroke. While this approach to facilitate functional recovery may not be appropriate in humans, it does suggest that increased responsiveness to therapy can be achieved by reestablishing a period of enhanced neuroplasticity.

Noninvasive brain stimulation techniques, such as transcranial direct current stimulation (tDCS), are novel approaches that may be able to facilitate neuroplasticity. It is thought that tDCS is capable of altering the level of intrinsic postsynaptic activity depending on the direction of current flow [13,14]. When applied to the primary motor cortex (M1), anodal tDCS increases cortical network excitability, and cathodal tDCS decreases cortical network excitability. Changes in excitability induced by tDCS are thought to be mediated by long-term potentiation and long-term depression-like synaptic plasticity [13,14]. Several studies have demonstrated functional improvements in people with stroke, following plasticity protocols applied to the lesioned M1 [15,16]. However, recent reviews highlight that, at the group level, tDCS does not provide additional benefits to therapy [17]. Upon further investigation, it appears responses can be highly variable among individuals, suggesting this is not a one-size-fits-all treatment. Several factors are known to influence the response to tDCS, including properties of the stimulated brain network, genetics, and endogenous cortisol levels [18,19]. Recently, we demonstrated that a measure of connectivity of the stimulated network was a strong predictor of response to anodal tDCS in healthy adults [20]. Using electroencephalography (EEG), we found that connectivity between electrodes overlying the stimulated M1 and the ipsilateral parietal cortex in the high beta frequency (20-30 Hz) predicted 69% of variability in the neuroplastic response to anodal tDCS using a leave-one-out and predict analysis. Along similar lines, connectivity of the stimulated ipsilesional motor network in alpha frequency (8-13 Hz) was strongly associated with the change in corticospinal excitability following tDCS in people with stroke [21].

Further evidence of the role that functional brain networks may play in modulating response to tDCS is available from other clinical populations. For example, in people with fibromyalgia, connectivity among the thalamus, posterior insula, motor cortex and sensory cortex was a marker of better analgesic response following tDCS applied to M1 [22]. Similarly, responses to tDCS applied to the dorsolateral prefrontal cortex in people in a minimally conscious state were associated with connectivity between the dorsolateral prefrontal cortex and inferior frontal gyrus [23]. It may be that the connectivity of the network targeted by tDCS can be a useful predictor of responsiveness to brain stimulation therapy. Indeed, this may be even more critical following a stroke, where damage as a result of the lesion can interrupt functional connectivity [24].

The primary objective of this study is to determine whether connectivity of the cortical target for tDCS modulates responses to this intervention in people with stroke. The secondary objectives of this study are to determine whether facilitatory tDCS applied to the ipsilesional hemisphere in combination with an upper limb exercise program provides greater behavioral improvement compared with sham stimulation and to determine whether additional neurophysiological characteristics, such as lesion size, cortical excitability, and white matter integrity, modulate the responsiveness to tDCS. We hypothesize that the response to anodal tDCS will be variable; participants who have greater functional connectivity of the ipsilesional motor network will have stronger responses to the stimulation as shown by a greater increase in upper limb function following the intervention period. Outcomes from this study will have important implications for the clinical translation of tDCS in stroke rehabilitation. The ability to select people who will respond to this therapy could substantially improve the clinical translation of this treatment approach.

## Methods

### Study Design

The SPIRIT (Standard Protocol Items: Recommendations for interventional trials) recommendations were referenced when developing this protocol. This protocol has been registered in the Australian and New Zealand Clinical Trials Registry (ACTRN12618000443291). This study is a double blind, randomized controlled trial, with 2 parallel groups. Both outcome assessors and participants will be blind to allocation. Randomization will be performed using a computerized sequence generation by an external researcher. As our primary research aim is to investigate the brain connectivity of participants allocated to the active treatment group, the allocation will be weighted 2:1 toward the active treatment group. A sham treatment group will be used as a comparator to demonstrate the effectiveness of this intervention at the group level and to demonstrate that brain connectivity is not associated with response to sham tDCS.

The study protocol has been approved by the University of South Australia Human Research Ethics Committee (application identification 0000036781; approved May 19, 2017). Recruited participants will provide written informed consent in accordance with the World Medical Association Declaration of Helsinki.

### Participants and Recruitment

Stroke participants will be recruited from the community by several strategies including placing information at local acute and tertiary hospitals, advertising through relevant charities and community services (eg, Stroke Foundation Australia and Stroke South Australia), speaking with local stroke survivor support groups, and advertising through social media. Textboxes 1 and 2 present the inclusion and exclusion criteria.

### Sample Size

Our primary aim is to determine the characteristics of the sensorimotor network at baseline that may predict the responsiveness to anodal tDCS in people with stroke. Therefore, our sample size calculation was based on pilot data of 10 people with stroke where we observed a medium to large effect size (r=0.56) for a correlation between baseline high beta frequency connectivity and change in cortical excitability following anodal tDCS [21]. Using this effect size with alpha set at .05 and power of 95%, we determined a total sample of 47 would be required. However, we will aim to recruit a total sample of 68, given our methodological approach of using unequal group allocation (additional 12%) and the nature of the home-based treatment with a longer follow-up study period (allowing 30% dropout). This will result in 45 participants in the active treatment group and 23 participants in the control group.

### Experimental Protocol

**Figure 1:**
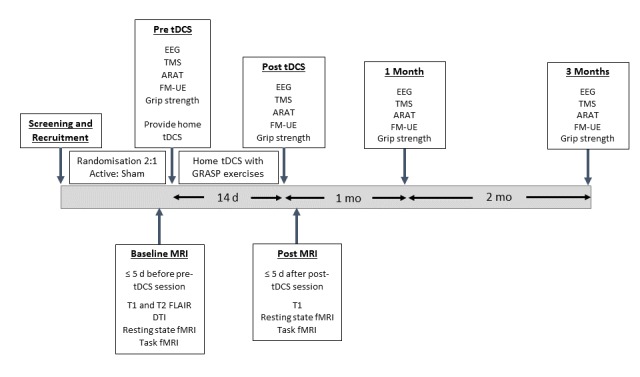
The schematic diagram of the experimental sessions. tDCS: transcranial direct current stimulation; EEG: electroencephalography; TMS: transcranial magnetic stimulation; ARAT: action research arm test; FM-UE: Fugl-Meyer Upper Extremity; GRASP: Graded Repetitive Arm Supplementary Program; MRI: magnetic resonance imaging; FLAIR: fluid-attenuated inversion recovery; DTI: diffusion tensor imaging; fMRI: functional magnetic resonance imaging.

Participants will attend 6 experimental sessions, as outlined in Figure 1. Session 1 will be conducted at the Clinical Research and Imaging Centre (Dr Jones and Partners, South Australian Health and Medical Research Institute) where magnetic resonance imaging (MRI) sequences will be performed to obtain structural, diffusion, and functional images. Session 2 will be conducted within 5 days of the initial MRI scan at the University of South Australia, City East Campus, Clinical Trials Facility. Participants will be encouraged to attend this experimental session with a supportive family member, friend, or carer. In this session participants will undergo baseline neurophysiological and behavioral outcome assessments and will be provided with a home tDCS kit (NeuroConn DC-Stimulator Mobile, NeuroConn GmbH, Ilmenau, Germany). Participants and their support person will be trained in the use of the home tDCS, iPad, and the Graded Repetitive Arm Supplementary Program (GRASP) exercises. In addition, information sheets will be provided for the use of the home tDCS equipment and the iPad. This information will detail the correct use of equipment, including locating the correct spot for tDCS electrode position, which will be marked on the scalp with a permanent marker. To facilitate training, the first of 14 treatments will be undertaken at the Clinical Trials Facility under the supervision of a research staff member. An iPad will be provided to each participant to facilitate monitoring of home tDCS using a telehealth platform. Each iPad will be preloaded with a videoconferencing platform (Skype), allowing the research staff to monitor the tDCS set-up and use across the intervention period.

Experimental sessions 3, 5, and 6 are respectively performed immediately, 1 month and 3 months following the final home tDCS treatment. During these sessions, participants will undergo neurophysiological and behavioral outcome assessments. Experimental session 4 is a follow-up MRI session and will occur within 5 days of the final tDCS treatment.

**Inclusion criteria**

- Aged ≥18 years of age
- At least 6 months after the first ever stroke with motor impairment
- Measurable impairment of the upper limb (Fugl-Meyer upper extremity of <62 out of 66)
- Supportive family, friends, or carers willing to actively assist and motivate across the 2-week intervention
- Active wrist extension of, at least, 5°
- Active index finger flexion of, at least, 10°
- Modified Ashworth scores of <4 for the affected elbow, wrist, and metacarpal phalangeal joints

**Exclusion criteria**

- Transcranial magnetic stimulation and transcranial direct current stimulation safety exclusion criteria as per international guidelines [25]
- Contraindications for magnetic resonance imaging
- Self-reported neglect, apraxia, or shoulder pain (>4 out of 10 on pain visual analog scale) that would affect the ability to undertake a 1-hour upper limb exercise program.
- Language or cognitive impairment that would limit the ability to communicate with the research team by videoconference
- Participation in a concurrent research study or clinical program for upper limb rehabilitation.

### Intervention

#### Graded Repetitive Arm Supplementary Program

All participants will be provided with a home exercise program using the GRASP [26]. The GRASP protocol consists of a booklet with detailed descriptions of multiple exercises for strength, range of motion, fine motor, and goal-directed activities targeting the upper limb. The provided kit includes all required equipment to perform the exercise program. The GRASP protocol is a self-administered program with program levels (grades 1-3) individualized by a qualified occupational therapist, based on the level of impairment of the upper limb. The upper limb exercises will be performed for 1 hour per day over a 2-week period (14 sessions).

#### Transcranial Direct Current Stimulation

Participants will also be provided with a home tDCS kit, which will be preprogrammed for 14 sessions of active or sham stimulation using a study code to facilitate research staff blinding. Participants will be unable to modify, or observe, any settings of the home tDCS devices. To initiate stimulation, participants will simply position electrodes on the head and press the start button. Those randomized to the “active” arm of the study will receive tDCS while simultaneously performing the GRASP exercises. TDCS will be delivered for 20 minutes and occur at the same time that a participant is undertaking the 1-hour GRASP program. Therefore, the initial 20 minutes of the intervention will involve both tDCS and the GRASP, with the remaining 40 minutes involving only GRASP exercises. The electrodes will be positioned with the anode over the ipsilesional M1 and cathode over the contralateral supraorbital region. TDCS will be applied at an intensity of 1 mA for 20 minutes daily for 2 weeks (total of 14 sessions) at home. Stimulation will be ramped up from 0 mA to 1 mA over the first 30 seconds and down from 1 mA to 0 mA over the final 30 seconds.

Participants randomized to the “sham” arm of the study will receive sham tDCS while undertaking the 1-hour individualized GRASP exercises. Electrodes will be positioned in the same location as the active tDCS group. Sham tDCS mimics the sensation of stimulation without changing cortical excitability. The protocol for sham tDCS will ramp the current up from 0 mA to 1 mA over the first 30 seconds before ceasing for the following 19 minutes and 30 seconds. This approach has been shown to provide an effective sham stimulation [27].

#### Compliance Monitoring

Several strategies will be used to monitor protocol compliance and ensure the correct use of home tDCS (for a summary see Textbox 3). The number of completed tDCS sessions and daily duration of GRASP will be recorded in a treatment diary.

#### Adverse Events and Assessment of Blinding

At the completion of the 2-week tDCS intervention, participants will be asked to complete a questionnaire to identify any adverse events and establish whether participant blinding was successful. In accordance with current recommendations [28], we will ask participants to rate on a scale of 1-4 (1, absent; 2, mild; 3, moderate; 4, severe) the presence of the following symptoms: headache, neck pain, scalp pain, tingling, itching, burning sensations, skin redness, sleepiness, trouble concentrating, acute mood change, and other symptoms. In addition, we will ask participants to what extent they believe the reported symptoms were related to using tDCS (1, none; 2, remote; 3, possible; 4, probable; 5, definite). Furthermore, we will ask participants to indicate whether they believe they received active stimulation (yes or no) to determine the effectiveness of blinding.

### Outcome Measures

Textboxes 4 and 5 summarize the independent and dependent variables to address the primary research question for this study. Participant demographics and clinical characteristics including age, gender, handedness [29], and time since stroke will be recorded and compared between the active and sham groups.

#### Assessments of Upper Limb Function

The primary outcome measure for this study is a change in upper limb impairment as measured with the Fugl-Meyer upper extremity (FM-UE) assessment. The FM-UE is a commonly used, validated, and reliable measure of sensorimotor impairment [30]. The FM-UE is considered as one of the most comprehensive quantitative measures of motor impairment following stroke.

In addition, upper limb function will be assessed with the action research arm test (ARAT) and grip strength. The ARAT is a valid and reliable measure of hemiplegic upper limb function [31]; it provides a quantitative measure of upper limb function for domains of grip, grasp, pinch, and gross arm movement. Grip strength is associated with motor cortical output and motor recovery [32]. We will measure grip strength using a hand dynamometer (SH5001 Saehan Hydraulic Hand Dynamometer, Saehan Co, Masan, Korea). The best (maximal) grip of 3 attempts will be recorded.

**Strategies to facilitate protocol compliance.**

- *Support person*: A support person will attend training for home transcranial direct current stimulation (tDCS) and graded repetitive arm supplementary program (GRASP) exercises to assist, and motivate, participants as required across the intervention.
- *Initial treatment under supervision*: The first treatment will be completed under supervision to ensure the correct use of tDCS.
- *TDCS electrode position marked on scalp*: The correct positioning of tDCS electrodes will be marked on the scalp with a permanent marker to facilitate home application.
- *Information sheet*: Step-by-step instructions for the use of the home stimulator.
- *Videoconference*: In real time, confirm correct tDCS usage, provide motivation, and progress individual exercise programs or GRASP grade. Videoconferences will occur every second day at a minimum and more frequently if required.
- *Exercise diary*: Record daily completion of tDCS and GRASP. Includes recording duration of GRASP therapy, motivation, fatigue, and perceived exercise difficulty.

**Dependent variables**

- Primary
  - Change in upper limb impairment (Fugl-Meyer Upper Extremity)
- Secondary
  - Change in the upper limb function (action research arm test)
  - Change in the upper limb strength (grip strength)
  - Change in the corticospinal excitability obtained from motor-evoked potential amplitude

**Independent variables**

- Functional connectivity
  - Task functional magnetic resonance imaging
  - Resting state functional magnetic resonance imaging
  - Electroencephalography
- Structural connectivity
  - Fractional anisotropy index of the corticospinal tract obtained from diffusion tensor imaging
- Anatomical
  - Lesion volume
- Demographics and clinical characteristics
  - Age
  - Gender
  - Time since stroke
  - Affected hemisphere based on hand dominance

Outcome assessors will be blind to group allocation and have completed training for FM-UE and ARAT assessments through the University of California Irvine. Training outcome assessors with this approach has been shown to improve accuracy and reduce variance of the FM-UE and ARAT [33,34].

### Neurophysiological Testing

#### Electroencephalography

Functional connectivity between brain regions will be assessed with high-density EEG. Three minutes of EEG will be acquired using an ASA-lab EEG system (ANT Neuro, Enschede, the Netherlands). Participants will be fitted with an ANT Waveguard cap with 64 sintered Ag-AgCl monopolar electrodes in standard 10-10 positions. Signals will be sampled at 2048 Hz, amplified 20X, filtered (high pass, DC; low pass, 553 Hz) and online referenced to CPz. During data recording, participants will be seated in a comfortable chair in a quiet room. Standardized instructions will be delivered to each participant asking them to relax during the 3 minutes of data recording, keep their eyes open, refrain from speaking or moving, maintain their gaze toward a fixation point straight ahead at eye level and not actively engage in any cognitive or mental tasks. Impedance will be kept <5 kΩ while recording.

Artifact rejection will be performed prior to analysis using independent component analysis. Nonphysiological artifacts will be identified using an automated and objective method to remove assessor bias [35]. Preprocessed data of participants with a right hemisphere lesion will then be flipped about the midline so that all lesions appear in the left hemisphere. Functional connectivity between electrodes will be determined using the debiased weighted phase lag index, which is a conservative estimate of connectivity based on phase consistency and biasing against zero phase lag relationships, limiting the detection of spurious measures of connectivity [36]. Regions of interest will include a seed approximating the ipsilesional M1 (C3) and clusters of electrodes approximating the ipsilesional premotor (F5, F3, FC5, and FC3), ipsilesional parietal (CP3, CP5, P3, and P5), and contralesional M1 (C4). Frequency bands of interest are the alpha band (8-15 Hz) and beta band (16-31 Hz) as they are associated with sensorimotor function [20,37,38]. For a given frequency, a debiased weighted phase lag index value of 1 indicates maximal phase coupling, whereas a value of 0 indicates no phase coupling. Connectivity analyses will be performed in MATLAB 9.2.0 (MathWorks, Inc) using both EEGLAB [39] and FieldTrip toolboxes [40].

#### Transcranial Magnetic Stimulation

Single-pulse transcranial magnetic stimulation will be used to quantify corticomotor excitability of the ipsilesional M1. Monophasic (posterior to anterior current flow) transcranial magnetic stimulation pulses will be delivered with a Magstim 200 stimulator (Magstim, Whitland, United Kingdom) via a figure-of-eight coil (70-mm wing diameter). The coil will be placed tangentially over the scalp with the handle pointing 45° posterolateral. Surface electromyography will be used to record motor-evoked potentials (MEPs) from the first dorsal interosseous muscle of the paretic hand with electrodes positioned in a belly-tendon montage. Suprathreshold stimuli will be delivered over the ipsilesional hemisphere to identify the optimal position for evoking an MEP from the first dorsal interosseous muscle of the paretic hand. For participants where MEPs cannot be evoked even at maximal stimulator output as a result of the stroke, we will document that a measure of corticospinal excitability was not obtainable at that experimental session. For participants where MEPs can be evoked, the optimal site will be marked on the scalp using a felt-tip marker to ensure consistent coil placement during subsequent data collection. Resting motor threshold will then be determined and is defined as the minimum stimulus intensity required to evoke an MEP of at least 50 μV in at least 5 of 10 consecutive trials. Thirty stimuli, not contaminated by pre-stimulus electromyography, will then be obtained at 120% resting motor threshold (interstimulus interval 4.5-5.5 sec) with the average, peak-to-peak amplitude, determined as a reliable measure of corticospinal excitability [41].

#### Magnetic Resonance Imaging

MRI will be performed at the Clinical Research and Imaging Centre located at the South Australian Health and Medical Research Institute with a Siemens 3T MAGNETOM Skyra scanner (Siemens, Erlangen, Germany). Standard MRI safety screening will be performed to ensure included participants are safe for MRI. At the preintervention MRI session, the imaging protocol will have a duration of 45 minutes and include T1 MPRAGE and T2-weighted fluid-attenuated inversion recovery images, diffusion tensor imaging, resting state, and task functional MRI (fMRI). At the postintervention MRI session, the imaging protocol will have a duration of 30 minutes and include T1-weighted images, resting state fMRI, and task fMRI.

The imaging protocols are as follows: T1-weighted images (MPRAGE, voxel 1 mm × 1 mm × 1 mm, repetition time (TR)=2300 ms, echo time (TE)=2.98 ms, flip angle=9°), T2-weighted fluid-attenuated inversion recovery images (voxel 1 mm × 1 mm × 1 mm, TR=5000 ms, TE=393 ms), diffusion MRI (voxel 2 mm × 2 mm × 2 mm, TR=4200 ms, b-value=0 and 2000 s mm), resting state fMRI (voxel 2.4 mm × 2.4 mm × 2.4 mm, TR=735 ms, TE=36 ms, 2 repeats of 6-min duration, 490 measurements for each), and task fMRI (voxel 2 mm × 2 mm × 2.5 mm, TR=3000 ms, TE=30 ms, 4.44-min duration). During the task fMRI, participants will be presented with a visual cue to squeeze a stress ball in their paretic or nonparetic hand, with blocks alternating every 30 seconds and repeated 4 times per hand.

Preprocessing and statistical analyses of MRI data will be carried out using tools from the FMRIB Software Library [42]. For all voxel-wise analyses, images from participants with lesions of the right hemisphere will be flipped about the midline after registration to standard space so that all lesions appear in the left hemisphere. fMRI data will be preprocessed and analyzed using the FMRIB Expert Analysis Tool [43]. Preprocessing steps will include high-pass temporal filtering at 0.01 Hz, spatial smoothing, motion correction, and removal of nonbrain tissue. Task fMRI data will be analyzed with a boxcar regressor, which will model task and rest blocks for first-level statistical maps for each participant. Higher level mixed-effects analysis will then be run using FMRIB Local Analysis of Mixed Effects [43] to test correlations with improvement in clinical scores and compare activation maps across groups.

For resting state fMRI, nuisance regressors of no-interest (cerebrospinal fluid, white matter, head motion, and physiological noise) will be modeled and removed. We will then calculate the mean time course of the blood-oxygenation level dependent signal in all voxels of the ipsilesional M1 region of interest. This time series will then be entered separately as an explanatory variable in the general linear model to determine for each participant the voxels where blood-oxygenation level dependent signal is temporally correlated with the ipsilesional M1. Connectivity between the ipsilesional M1 and other regions of interest (contralesional M1, ipsilesional dorsal premotor cortex, ipsilesional ventral premotor cortex, ipsilesional supplementary motor area, and ipsilesional posterior parietal cortex) will be determined with a Pearson’s correlation coefficient. Connectivity of the ipsilesional M1 will be compared before and after tDCS with a general linear model.

Structural connectivity will be analyzed with the FMRIB Diffusion Toolbox [44]. For each participant, the mean fractional anisotropy (FA) of the posterior limb of the internal capsule will be determined. An asymmetry index will be calculated as follows: FA = (FA–FA) / (FA+FA).

Lesion volume will be defined by manually tracing the lesion in FLS view. Lesions will be traced from each participant’s T1-weighted image using the coregistered T2-weighted image as a reference for lesion extension.

### Statistical Analysis

Normality of data will be confirmed using Shapiro-Wilk normality tests. Where required, data will be normalized using transformations or nonparametric statistics applied. Participants’ demographics and clinical characteristics will be compared between active and sham groups. The effect of the intervention on behavioral and neurophysiological outcome measures will be investigated with a 2 Group (active, sham) × 4 Time Point (Baseline, Postintervention, 1-Month Follow-up, 3-Month Follow-up) repeated measures analysis of variance. Independent variables (Textbox 5) will be correlated with the primary outcome measure for response to anodal tDCS (change in upper limb impairment measured with the UE-FM). Where appropriate, regression models will be generated using those independent variables significantly correlated with change in upper limb impairment to identify a combination of measures associated with response to anodal tDCS. Any regression models generated will be compared using the Bayesian information criteria [45]. Where appropriate, the predictive capacity of the generated model will be investigated using a leave-one-out cross-validation. This cross-validation will be performed on participants allocated to both active and sham treatment groups to demonstrate that the predictive model is specific to the stimulation group. Statistical testing will be performed using SPSS (IBM Co, version 24.0) and significance level will be *P* ≤.05.

## Results

As of April 2018, 11 participants have been enrolled in the study with 5 beginning experimental testing. It is anticipated that the final participant enrollment will occur in December 2018, with data collection completed in March 2019. At the conclusion of the study, results will be disseminated through publication in scientific journals and conference presentations.

## Discussion

Adjuvant therapies, such as tDCS, are critical to improving the potential for motor function recovery following stroke. To date, the response to tDCS has proved highly variable, and this has limited clinical translation. This is likely due, at least in part, to stimulation being applied without consideration of individual motor network characteristics. This study will be a significant step forward in the development of precision approaches for the use of brain stimulation in stroke rehabilitation. This will be achieved by providing evidence for biomarkers of brain connectivity to selectively apply tDCS to those stroke patients who will benefit most. Future work could lead to individualized brain stimulation protocols based on motor network connectivity and clinical presentation. This body of work has the potential to enhance functional outcomes for a population that presents a significant social and economic burden and are desperate for improved rehabilitation services.

## Abbreviations

ARAT: action research arm test
EEG: electroencephalography
FA: fractional anisotropy
fMRI: functional magnetic resonance imaging
FM-UE: Fugl-Meyer upper extremity
GRASP: graded repetitive arm supplementary program
M1: primary motor cortex
MEP: motor-evoked potential
MRI: magnetic resonance imaging
tDCS: transcranial direct current stimulation

## Appendix

Multimedia Appendix 1 Peer-review report from the Sylvia and Charles Viertel Charitable Foundation.
