# Supplementary material for: Connectivity as a Predictor of Responsiveness to Transcranial Direct Current Stimulation in People with Stroke: Protocol for a Double-Blind Randomized Controlled Trial
Source: JMIR Res Protoc. 2018 Oct 18;7(10):e10848. doi: 10.2196/10848 (PMC6231838; doi:10.2196/10848)
Supplement: Multimedia Appendix 1 [file resprot_v7i10e10848_app1.pdf]

CIA Name Doctor Brenton Hordacre

Application Title Characterising motor network connectivity to improve application of non-invasive brain stimulation in stroke

External Assessor 1

Scientific Quality

This is a 4 year project, wherein 75 stroke patients will be allocated to one of 3 arms: sham stimulation, anodal ipsilesional stimulation, or cathodal contralesional stimulation, using transcranial direct current stimulation (tDCS). The proposal was difficult to read and follow at times, and some of the language was very diffuse.

There are 2 aims and 6 hypotheses. Aim 1 is to determine if measures of motor network connectivity are predictive of neurophysiological and behavioural responses to combined tDCS and upper limb exercise, and Aim

2 to determine if functional assessments of the upper limb are predictive of motor network connectivity. They plan to measure motor connectivity via EEG coherence (resting state EEG). A sample size calculation is included but statistical design is only briefly discussed.

I have the following questions:

1. There is no strong anatomical hypothesis to your connectivity analyses. Does this represent the relative bluntness of the EEG measures?

2. H 1.3 “interhemispheric connectivity of the lesioned and non-lesioned motor cortex” – by definition doesn’t interhemispheric imply the connections between the 2 hemispheres?

3. Define a strong response as hypothesised. You often use the terms strengthened and weakened yet I cannot establish what metrics you are basing this on.

4. What do you mean by behavioural capacity? Do you mean strength? Functionality? Defined how?

5. You make no mention of repeating functional measures. Will you be repeating the NIHSS and Rankin to see if there are global changes?

6. Will you be doing any measures of lower limb function to see whether there are secondary effects? Similarly, any measures of cognition?

7. What reaction time task are you using?

External Assessor 2

01. Scientific Quality

The literature reviewed in this proposal provides a strong rationale for the aims and hypothesis of the proposed study and the overall methodology is sound. Clinical links will facilitate recruitment. Issues that need clarification:

1. There is a mismatch between the aims and hypothesis and study design. There is no specific aim to compare response between the two stimulation and sham sites. The inclusion of 3 groups seems reasonable and from the analysis it appears that the researchers are interested in this question. It would be useful to have the aims and hypothesis of any group comparisons explicitly stated.

2. It is unclear how sample size is calculated; it is based on effect size from preliminary studies and other work, but actual effect size is not given. It is also not clear whether this calculation is sufficient to determine differences between groups or strength of associations. Given the heterogeneous nature of stroke and the likely high variability between people, this seems like a small sample size if the researchers are interested in between group differences. While the intervention period is short, there is also likely to be some drop out during the follow up period which does not seem to have been taken into account.

3. While the researchers have collected some data in healthy subjects and in 4 people with stroke (eg EEG, tDCS) , the proposed protocol has not been trialled so the feasibility of delivering the proposed intervention has not been demonstrated. For example, there is no evidence that the in home tDCS has been used with people with stroke so it is not clear whether this can be delivered as planned

Other issues include whether any ongoing therapy may confound results; who is delivering the GRASP; is only a single intervention session with the physiotherapist. Clarify who on the research team will be doing the assessments/ delivering the interventions (and which members of the team will be blinded and who won't be).

This is an innovative study that has potential to transform rehabilitation for people with stroke, and may have implications for other clinical groups. Of note, the technology and methodology used could be translated to a clinical or home based setting, which would facilitate translation.
